# EPH receptor B2 stimulates human monocyte adhesion and migration independently of its EphrinB ligands

**DOI:** 10.1002/JLB.2A0320-283RR

**Published:** 2020-04-26

**Authors:** Dianne Vreeken, Caroline Suzanne Bruikman, Stefan Martinus Leonardus Cox, Huayu Zhang, Reshma Lalai, Angela Koudijs, Anton Jan van Zonneveld, Gerard Kornelis Hovingh, Janine Maria van Gils

**Affiliations:** ^1^ Department of Internal Medicine Einthoven Laboratory for Vascular and Regenerative Medicine Leiden University Medical Center Leiden The Netherlands; ^2^ Department of Vascular Medicine Amsterdam Cardiovascular Sciences Amsterdam UMC University of Amsterdam Amsterdam The Netherlands

**Keywords:** atherosclerosis, EPH signaling, monocyte‐endothelial interaction

## Abstract

The molecular basis of atherosclerosis is not fully understood and mice studies have shown that Ephrins and EPH receptors play a role in the atherosclerotic process. We set out to assess the role for monocytic EPHB2 and its Ephrin ligands in human atherosclerosis and show a role for EPHB2 in monocyte functions independently of its EphrinB ligands. Immunohistochemical staining of human aortic sections at different stages of atherosclerosis showed that EPHB2 and its ligand EphrinB are expressed in atherosclerotic plaques and that expression proportionally increases with plaque severity. Functionally, stimulation with EPHB2 did not affect endothelial barrier function, nor did stimulation with EphrinB1 or EphrinB2 affect monocyte‐endothelial interactions. In contrast, reduced expression of EPHB2 in monocytes resulted in decreased monocyte adhesion to endothelial cells and a decrease in monocyte transmigration, mediated by an altered morphology and a decreased ability to phosphorylate FAK. Our results suggest that EPHB2 expression in monocytes results in monocyte accumulation by virtue of an increase of transendothelial migration, which can subsequently contribute to atherosclerotic plaque progression.

## Graphical Abstract

EPHB2 expression in monocytes promotes monocyte accumulation by increasing monocyte adhesion and transendothelial migration; subsequently, this can contribute to atherosclerotic plaque progression.

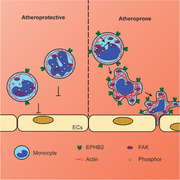

**Abbreviations**
AKT: protein kinase B
ASO: antisense oligonucleotide
CCR2: C‐C chemokine receptor 2
CD‐: Cluster of differentiation
CVD: cardiovascular disease
ECIS: electric cell‐substrate impedance sensing system
EPH: erythropoietin‐producing hepatocellular receptor
Ephrin: EPH receptor interacting protein
FAK: focal adhesion kinases
HBSS++: HBSS with calcium and magnesium
HUVEC: human umbilical vein endothelial cell
ITGB1: integrinβ‐1
MCP‐1: monocyte chemotactic protein 1
NGC: neuroimmune guidance cue
PBS: phosphate buffered saline
Y: Tyr, Tyrosine

## 1 INTRODUCTION

Cardiovascular disease (CVD), caused by atherosclerosis, remains the leading cause of death.^1^ Despite the use of CVD risk lowering agents patients still suffer from CVD events, which suggest that additional, hitherto unaddressed, factors are involved.^2^, ^3^ Recently, a long‐assumed role for inflammation in the atherosclerotic process has been proven,^4^, ^5^, ^6^ indicating that other pathophysiologic processes may play a role in CVD development. Unraveling novel players in the complex atherosclerotic process may ultimately result in novel targets for therapies to address the endemic burden of atherosclerosis.

Neuroimmune guidance cues (NGCs) are a group of proteins, consisting of 4 families of guidance molecules and their receptors, Netrins, Slits, Ephrins, and Semaphorins. These cues were originally found to play a crucial role in the process of axon growth. However, NGCs have also been shown to play a role in atherosclerosis, as NGCs also regulate the development of the vascular system, maintain the physiological function of endothelial cells, and play an important role in immune cell trafficking.^7^, ^8^, ^9^ The endothelial expression of several NGCs has been shown to differ between athero‐resistant and athero‐prone aortic regions. Moreover, NGCs have been implicated in leukocyte adhesion and migration, which implies that NGCs are crucial in the initial steps of atherogenesis.^10^ Specifically, several studies have shown that members of the Ephrin family are involved in atherosclerosis related processes.^10^, ^11^, ^12^, ^13^, ^14^, ^15^

Erythropoietin‐producing hepatocellular receptors (EPHs) and their EPH receptor interacting protein (Ephrin) ligands comprise a large family of receptor tyrosine kinases with 14 EPH receptors and 8 Ephrin ligands that are both membrane bound. A special feature of the Ephrins and their receptors is that they can induce bidirectional signaling. Not only does binding of the ligand to the receptor induce signaling (forward signaling), but also receptor‐to‐ligand binding induces signaling (reverse signaling). Both forward and reverse Ephrin signaling impacts on a variety of signaling pathways that mostly converge to regulation of the cytoskeleton and therewith can influence processes such as cellular adhesion, migration, and vascular stability. Due to its role in a variety of cellular processes, deregulation of the Ephrins have been associated with several diseases, including atherosclerosis.^16^

Multiple Ephrins and EPH receptors have been found in human atherosclerotic plaques.^13^, ^14^, ^17^ In addition, the EPH receptor genes *EPHA2*, *EPHA8*, and *EPHB2* are located on chromosome 1 within region *1p34‐36*, which has been identified as a locus for myocardial infarction by a genome wide search for susceptibility genes for myocardial infarction.^18^ However, the functional role for Ephrins and EPH receptors in atherosclerosis is largely unexplored. We hypothesized that these molecules are expressed in human monocytes and endothelial cells, both culprit cell types in atherosclerosis, and contribute to atherogenesis. In this study, we identified the EPHB2 receptor and the ligands EphrinB1 and EphrinB2 as highly expressed Ephrin family members on monocytes and endothelial cells respectively. In addition, we showed increasing expression of both EPHB2 and EphrinB in progressing stages of atherosclerosis. Furthermore, we demonstrated an important EphrinB ligand‐independent role for EPHB2 in the atherosclerotic process, by promoting monocyte adhesion through phosphorylation of focal adhesion kinases (FAK).

## 2 MATERIALS AND METHODS

### 2.1 Database

We evaluated which NGCs are expressed by either monocytes or endothelial cells by means of the GENEVESTIGATOR^19^ software. All published data on the Affymetrix Human Genome U133 Plus 2.0 Array (HG0U133 Plus 2.0/GPL570) platform on human leukocyte, endothelial cell, or vascular smooth muscle cell gene expression were extracted and analyzed for Ephrin ligands and EPH receptor expression.

### 2.2 Immunohistochemistry/fluorescence of human tissue sections

The expression profiles of EPHB2 and EphrinB in human abdominal aortas at different stages of atherosclerosis were analyzed. The abdominal aorta segments used for this study were harvested during renal surgery. Use of this material is approved by the Medical and Ethical Committee of the Leiden University Medical Center (Leiden, the Netherlands). Approximately 3 cm of arterial material was fixed with formaldehyde and subsequently decalcified with Kristensen's solution to allow sectioning. The tissue was sliced in 5 mm segments and paraffin embedded. Tissue sections of 4 µm were prepared from each segment and each tissue block was classified for atherosclerotic stage using the revised classification of the American Heart Association.^20^ In sections with multiple lesions, grading was dictated by the most advanced lesion present.

Before staining, the slides were deparaffinized in 100% xylene and rehydrated in ethanol. Heat‐induced epitope retrieval was performed in citrate buffer (pH 6.0) for 20 min at 98°C. Next, nonspecific Ags were blocked with 1% BSA in TBS for 30 min, followed by incubation with goat‐anti‐EPHB2 (5 µg/ml, AF467, R&D Systems, Minneapolis, MN, USA) or mouse‐anti‐EphrinB (1.5 µg/ml, 37–8100, Thermofisher, Rockford, IL, USA) for 60 min. Slides were incubated with HRP‐labeled rabbit‐anti‐goat (1:2000, P0160, Dako, Glostrup, Denmark) or HRP‐labeled goat‐anti‐mouse (1:80, P0447, Dako, Glostrup, Denmark) secondary Ab for 60 min and counterstained with NovaRed Peroxidase (SK‐4800, Vector laboratories, Burlingame, CA, USA). Slides were covered with glycergel (C0563, Agilent, Glostrup, Denmark) or pertex (00801, Histolab, Västra Frölunda, Sweden) and a glass coverslip. For double staining, slides were incubated overnight with goat‐anti‐EPHB2 together with either mouse‐anti‐CD68 (1:100, MCA1815T, BioRad, Temse, Belgium) or mouse‐anti‐CD45 (1:100, MBS245401, MyBioSource, San Diego, CA, USA). After a 30‐min incubation with the secondary Abs alexa568‐labeled donkey‐anti‐goat and alexa488‐labeled donkey‐anti‐mouse (1:250, A11057 and A21202, Invitrogen Molecular probes, Eugene, OR, USA) slides were mounted with ProLong™ Gold Antifade mountant with DAPI (P36931, Thermo Fisher, Eugene, OR, USA).

Images were taken with the Pannoramic MIDI slide scanner and processed and quantified with HistoQuant software from 3DHistech. The investigator performing and scoring the grade of staining was blinded for the stage of atherosclerosis.

### 2.3 Primary cells, cell lines and media

#### 2.3.1 HUVEC isolation

Primary HUVECs were isolated from human umbilical cords obtained at the Leiden University Medical Center after written informed consent and ensuring that collection and processing of the umbilical cord was performed anonymously. The umbilical vein was flushed with PBS, using glass cannulas, to remove all remaining blood. Endothelial cells were detached by infusion of the vein with Trypsin/EDTA (1×) (BE02‐007E, Lonza, Verviers, Belgium) solution and incubation at 37°C for 15 min. After incubation, the cell suspension was collected and taken up in endothelial cell growth medium (EGM2 medium, C222111 supplemented with C39211, Promocell, Heidelberg, Germany) with 1% antibiotics. After flushing the umbilical vein once more with PBS, to ensure all detached cells are collected, cells were pelleted by centrifugation at 1200 rpm for 7 min. The cell pellet was dissolved in fresh EGM2 medium and cells were cultured on gelatin (1%) coated surfaces.

#### 2.3.2 THP1 cells

THP1 (TIB‐202, ATCC, Middlesex, UK) were cultured in RPMI 1640 medium (22409, Gibco, Paisley, UK) supplemented with 10% FCS, 1% l‐glutamine, 1% antibiotics (penicillin/streptomycin, 15070063, Gibco, Paisley, UK), and 25 nM β‐mercaptoethanol. Differentiation of THP1 cells was achieved by a 3‐day incubation with 100 nM PMA after which cells were cultured for another 5 days in normal growth medium.

#### 2.3.3 CD14^+^ PBMCs

PBMCs were isolated from buffy coats (Ethical Approval Number BTL 10.090) obtained after informed written consent by density gradient separation using Ficoll. CD14 Microbeads (130‐050‐201, Miltenyi Biotec, Bergisch Gladbach, Germany) and LS columns (130‐042‐401, Miltenyi Biotec, Bergisch Gladbach, Germany) were used for magnetic separation of CD14 positive monocytes. Isolated cells were kept in RPMI 1640 medium supplemented with 10% FCS, 1% l‐glutamine, and 1% antibiotics (penicillin/streptomycin). Cells were stimulated with 20 ng/ml M CSF (130‐093‐963, Miltenyi Biotec, Bergisch Gladbach, Germany) for 7 days to induce monocyte‐to‐Mϕ differentiation.

### 2.4 Transduction of THP1 cells

To achieve a knockdown of EPHB2 or EphrinB1, THP1 monocytes were transduced with lentiviral particles encoding shRNA against de coding region of EPHB2 or EphrinB1 (MISSION library Sigma–Aldrich, TRCN0000006424 or TRCN0000058656 respectively) or a mock. Selection of transduced cells was achieved using puromycin (3.33 µg/ml).

### 2.5 Barrier function assay

Endothelial barrier function analysis was performed with impedance‐based cell monitoring using the electric cell‐substrate impedance sensing system (ECIS Zθ, Applied Biophysics). ECIS plates (96W20idf PET, Applied Biophysics, Troy, NY) were pretreated with 10 mM l‐cystein and coated with 1% gelatin. Baseline resistance was measured over ≈1 h after which endothelial cells were added to the plate. Multiple frequency/time mode was used for the real‐time assessment of the barrier and monolayer confluence. After ≈24 h when a stable barrier was formed, endothelial cells were stimulated with 500 ng/ml of recombinant EPHB2 (5189‐B2, R&D Systems, Minneapolis, MN, USA).

### 2.6 Adhesion assay

THP1 cells with or without knockdown were labelled with 5 µg/ml Calcein AM (C3100MP, Molecular Probes, Eugene, OR, USA) and incubated on top of a monolayer of HUVECs for 30 min at 37°C. Non‐adhering cells were washed away by multiple washing steps with PBS after which the cells were lysed in Triton‐X 0.5% for 10 min. Fluorescence was measured at λex 485 nm and λem 514 nm. Each condition was performed in triplicate. In case of cell stimulation, THP1 cells were stimulated with 500 ng/ml recombinant EphrinB1 (7654‐EB, R&D Systems, Minneapolis, MN, USA) or EphrinB2 (7397‐EB, R&D Systems, Minneapolis, MN, USA) for 30 min before addition to the monolayer of endothelial cells.

### 2.7 Migration assay

Chemotaxis of THP1 monocytes was measured using a 24‐well Boyden chamber with a 5 µm pore size filter (734‐1573, Corning, Kennebunk, ME, USA) coated with 10 µg/ml fibronectin (F4759, Sigma, Saint Louis, MO, USA). Cell migration toward 10 ng/ml recombinant human MCP‐1 (279‐MC, R&D Systems, Minneapolis, MN, USA) and/or 500 ng/ml EphrinB1 or EphrinB2 was measured after 3 h. Cells were resuspended and counted in randomly selected fields for each well to determine the number of cells that had migrated into the lower chamber. Each condition was performed in triplicate.

### 2.8 Real‐time PCR

Total RNA was isolated using TRIzol and the RNeasy Mini Kit (74106, Qiagen, Hilden, Germany) according to manufacturer's instructions. Total RNA was reverse transcribed using M‐MLV Reverse Transcriptase Kit (M1701, Promega, Madison, WI, USA). RT‐PCR analysis was conducted using SYBR Select Master Mix (4472908, Applied Biosystems, Vilnius, Lithuania) and the forward and reverse primers as indicated in Supplementary Table 1. The PCR cycling conditions were: initial denaturation at 95°C for 10 min, followed by 40 cycles at 95°C for 15 s, 60°C for 30 s, and 72°C for 30 s, followed by a final extension step at 72°C for 10 min. mRNA expression was normalized to expression of GAPDH and expressed as fold change compared to untreated.

### 2.9 Immunoblot analysis

THP1 cells were washed with cold PBS and lysed in cold RIPA buffer (9806, Cell signaling, Danvers, MA, USA). After centrifugation of the samples at 14,000 rpm for 10 min at 4°C, protein concentration in the supernatant was measured using the Pierce BCA Protein Assay Kit (23255, Thermo Scientific, Rockford, IL, USA). Equal amounts of protein sample were denatured using DTT and heating at 95°C for 10 min followed by size separation on a 10% Mini‐PROTEAN gel (4561033, Biorad, Temse, Belgium). Proteins were transferred to PVDF membranes (1704156, Biorad, Temse, Belgium) using the Trans‐Blot Turbo system (Biorad) after which membranes were blocked in either TBST‐5% BSA (A2058, Sigma, Saint Louis, MO, USA) for phosphorylated proteins or TBST‐5% milk. Overnight incubation was performed with primary Abs against EPHB2 (0.5 µg/ml, AF467, R&D systems, Minneapolis, MN, USA), EphrinB (1:250, 37–8100, ThermoFisher, Eugene, OR, USA) p38 (1:1000, 9211, Cell signaling, Danvers, MA, USA), P‐p38 (1:1000, 9212, Cell signaling), p42 (1:1000, 9102, Cell signaling), P‐p42 (1:1000, 4376, Cell signaling), FAK (1:500, 3283, Cell signaling), P‐Y397 FAK (1:500, 3283, Cell signaling), P‐Y925 FAK (1:500, 3284, Cell signaling), Akt (1:1000, 4060, Cell signaling), P‐Akt (1:1000, 4691, Cell signaling), or GAPDH (1:5000, 5174S, Cell signaling). Incubation with HRP‐conjugated secondary Abs (1:5000, Dako, Glostrup, Denmark) and Western lightning ECL (NEL103001EA, PerkinElmer, Waltham, MA, USA) or SuperSignal Western Blot Enhancer (46640, ThermoFisher, Rockford, IL, USA) enabled us to visualize protein bands with the ChemiDoc Touch Imaging System (Biorad). Expression was quantified using ImageLab software (Biorad) and ImageJ software (http://rsbweb.nih.gov/ij/).

### 2.10 Immunofluorescence of cultured cells

Monocytes were incubated on fibronectin‐coated (10 µg/ml, F4759, Sigma, Saint Louis, MO, USA) flat bottom 96‐wells plates for 30 min, washed with PBS, fixed with 4% paraformaldehyde in HBSS modified with calcium and magnesium (HBSS++) for 10 min and permeabilized with 0.1% Triton X‐100 for 1 min. After a 30‐min blocking step with 2% Casein in HBSS++, wells were incubated with Phalloidin‐Rhodamine (1:200, P1951, Sigma, Saint Louis, MO, USA) for 1 h. Excess Phalloidin staining was washed off and cells were imaged using the ImageXpress (Molecular Devices) and cell area was quantified using MetaXpress (Molecular Devices).

### 2.11 Statistical analyses

Data was analyzed by unpaired two‐tailed *t*‐tests for 2 groups or with ANOVA and post hoc *t*‐tests by the Tukey method for multiple groups. *P*‐values of < 0.05 were considered statistically significant. All statistical analyses were performed with SPSS version 24 or Graphpad Prism 7.

## 3 RESULTS

### 3.1 Increased expression of EPHB2 and EphrinB in progressive human atherosclerotic lesions

**FIGURE 1:**
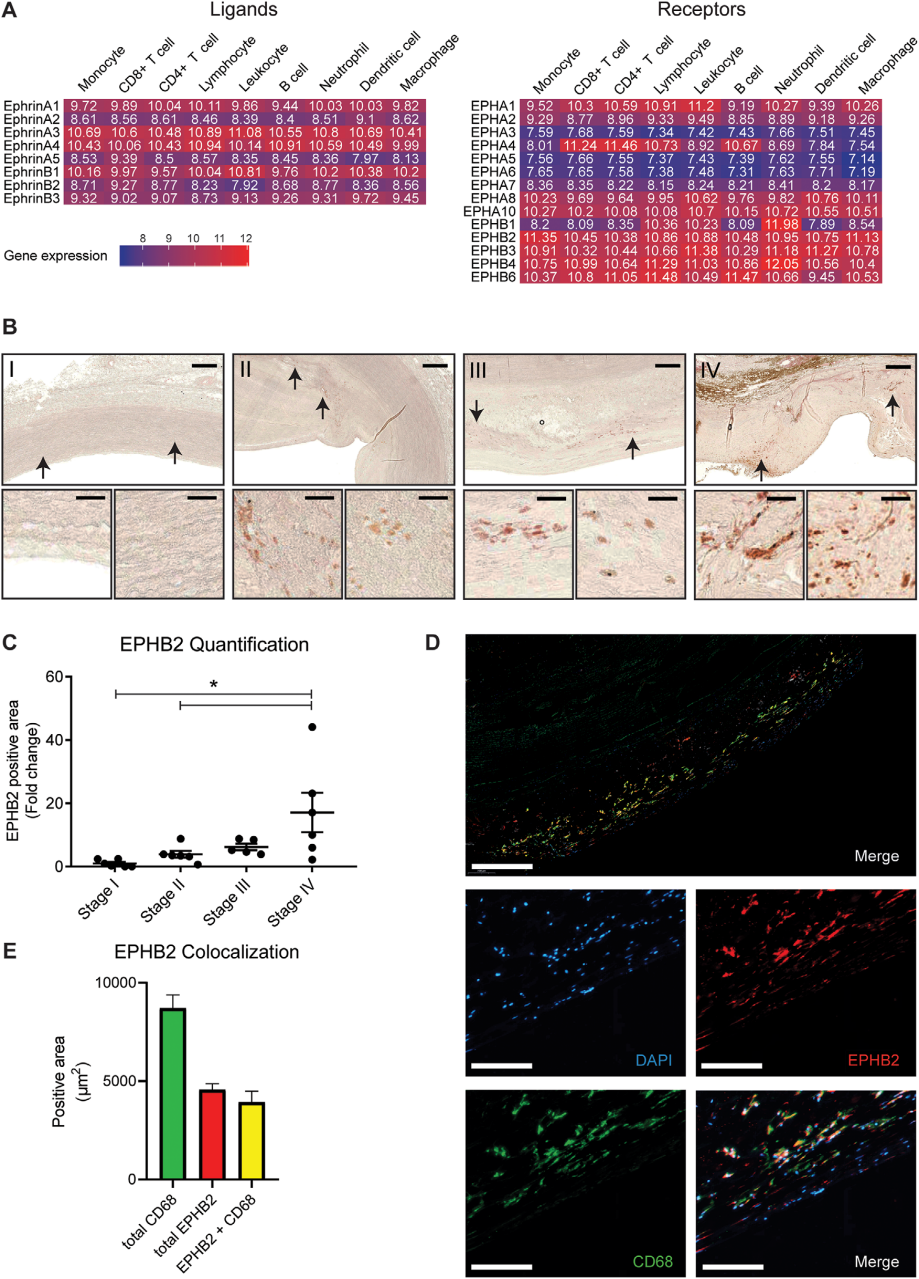
**Increased expression of EPHB2 in progressive human atherosclerotic lesions**. (**A**) Expression heatmap of Ephrin family ligands and their receptors in human leukocytes. Blue indicates lower and red higher expression. (**B and C**) Immunohistochemical staining for EPHB2 in human aortic sections in different stages of atherosclerosis. (**B**) Overview and and higher‐power magnification of the arrow‐indicated fields. Scale bars represent 350 and 50 µM, respectively. (**C**) Quantification of EPHB2 signal. Results are relative to stage I, set as 1. Mean ± sem of *n* = 6. ^*^ *P*  <  0.05. Stage I: Normal, Adaptive Intimal Thickening, Intima Xantoma; II: Pathologic intimal thickening, early fibroatheroma; III: Late fibroatheroma, thin cap fibroatheroma, ruptured plaque; IV: Healing rupture, fibrous calcifies plaque. (**D** and **E**) Immunofluorescent staining of EPHB2 (red), CD68 (green), and nuclei (blue) in stage IV human aortic sections. (**D**) Overview and zoom‐in pictures, scale bars represent 300 and 25 µm, respectively. (**E**) Quantification of fluorescent signal in plaque shoulder regions. Results are quantified as positive area in µm^2^. Mean ± sem of *n* = 6

Atherosclerosis is a systemic inflammatory disease, characterized by the accumulation of inflammatory cells in the vascular wall.^21^ Monocyte derived macrophages are key players in the lesion development.^22^ Therefore, we investigated the expression of Ephrin ligands and receptors in human monocytes and in atherosclerotic lesions. For this, we combined published data within the Affymetrix Human Genome U133 Plus 2.0 Array platform and determined the specific Ephrins and EPH receptors expressed by leukocytes relative to the averaged normalized expression (Fig. 1A). In general, all ephrin ligands were expressed moderately in human leukocytes, with the highest expression observed for *EphrinA1*, *EphrinA3*, *EphrinA4*, and *EphrinB1*. Expression of Ephrins differed minimally between leukocytes. In addition, the EPH receptors *EPHA1*, *EPHA2*, *EPHA8*, *EPHA10*, *EPHB2*, *EPHB3*, *EPHB4*, and *EPHB6* were moderately expressed in all leukocytes. Strikingly, *EPHA1* and *EPHA4* were more abundant in lymphocytes, while *EPHB2* was more abundantly expressed in human monocytes and macrophages. Based on this observation, an observed increase in *EPHB2* expression upon monocyte‐to‐Mϕ differentiation (Supplementary Fig. 1) and combined with the fact that the *EPHB2* gene is located on a myocardial infarction susceptibility locus,^18^ we hypothesized a role for monocytic EPHB2 in atherosclerosis development. To investigate this, immunohistochemical staining for EPHB2 was performed on 24 aortic specimens with varying stages of atherosclerosis, ranging from Stage I (Normal, Adaptive Intimal Thickening, Intima Xantoma) to Stage IV (Healing Rupture, Fibrous Calcifies Plaque). EPHB2 was near absent in normal vascular tissue (Fig. 1B and C, Stage I). However, expression was found to progressively increase with atherosclerotic lesion formation, up to a 17‐fold increase in stage IV (Fig. 1B and C). As EPHB2 expression was primarily observed in severe atherosclerotic lesions, we performed a double staining of EPHB2 with the leukocyte marker cluster of differentiation (CD)45 or the monocyte/Mϕ marker CD68 on Stage IV tissue sections to determine the contribution of immune cells to the observed increase in EPHB2 expression. Double staining with CD45 showed that around 25% of the CD45^+^ area was also positive for EPHB2 (Supplementary Fig. 2). Double staining of EPHB2 with CD68 revealed that around half of the area occupied by CD68^+^ cells was double positive for EPHB2 (Fig. 1D and E). Moreover, ≈80% of the EPHB2^+^ area was also positive for CD68, together indicating that most cells positive for EPHB2 were monocytes/macrophages.

**FIGURE 2:**
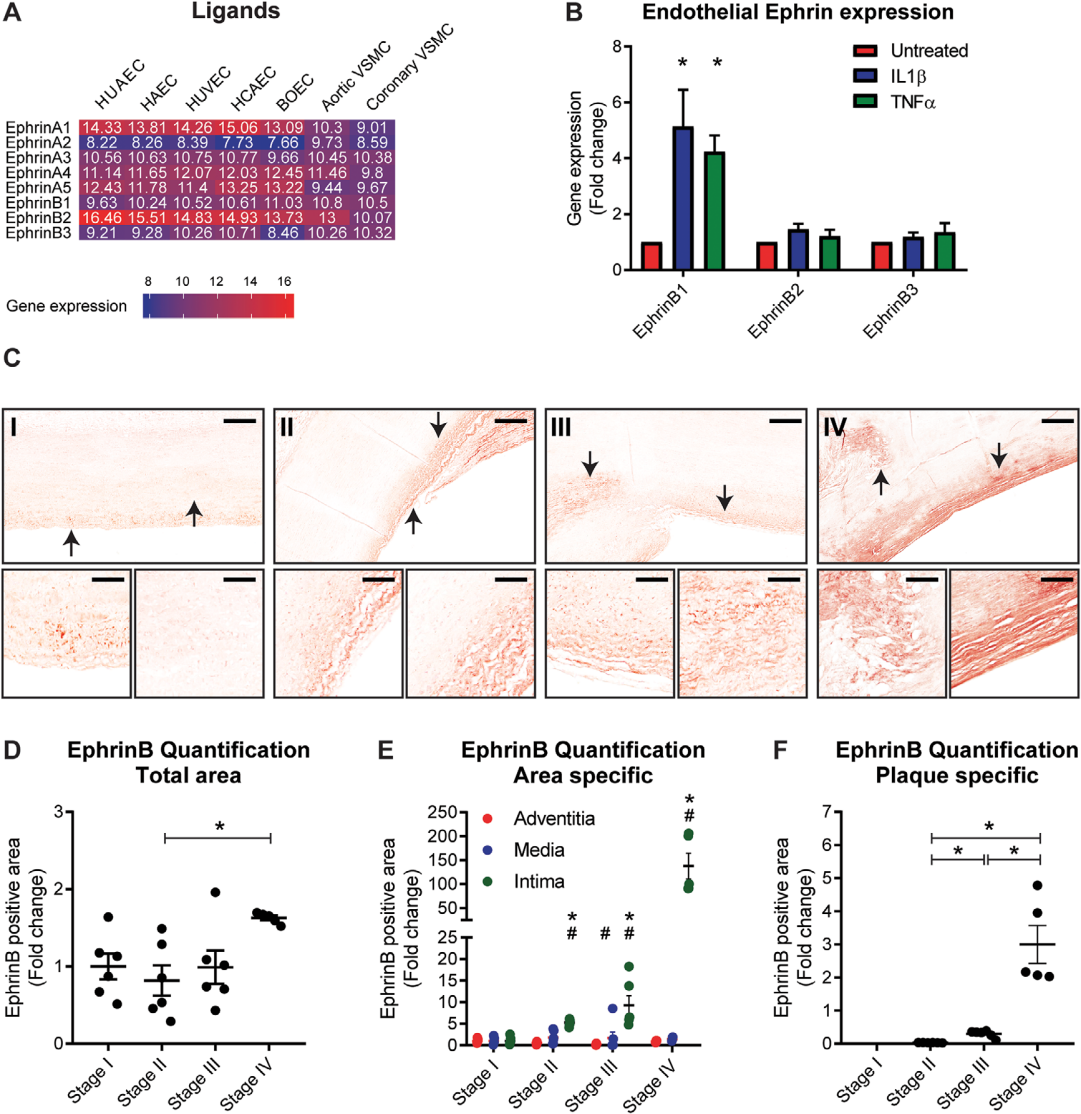
**Increased expression of EphrinB in progressive human atherosclerotic lesions**. (**A**) Expression heatmap of Ephrin family ligands in endothelial cells and vascular smooth muscle cells. Blue indicates lower and red higher expression. (**B**) EphrinB1/B2/B3 expression in HUVECs stimulated with 20 ng/ml IL‐1β or 10 ng/ml TNF‐α for 24 h. Results are relative to untreated cells, set as 1. Mean ± sem of *n* = 3. *P < 0.05. (**C**) Overview pictures of immunohistochemical staining for EphrinB in human aortic sections in different stages of atherosclerosis. Lower images show higher power magnification of the arrow‐indicated fields. Scale bars represent 350 and 50 µm, respectively. (**D–F**) Quantification of EphrinB signal on total area (**D**), adventitia, media, and intima regions (**E**) and total plaque area (**F**). Results are relative to stage I, set as 1. Mean ± sem of *n* = 6. (**D–F**) ^*^ *P* < 0.05 compared to indicated stages. (**E**) ^*^ *P* < 0.05 representing interstage variability within a region compared to stage I and ^#^ *P* < 0.05 representing intrastage variability between regions. Stage I: normal, adaptive intimal thickening, intima xantoma; II: pathologic intimal thickening, early fibroatheroma; III: late fibroatheroma, thin cap fibroatheroma, ruptured plaque; IV: healing rupture, fibrous calcifies plaque

It is well established that the recruitment and accumulation of monocytes is regulated by both chemoattractant signals and changes in the adhesive properties of the endothelium lining the vascular wall.^21^ Since we suspected an interaction of monocytic EPHB2 with Ephrin ligands expressed by the endothelial lining of the vessel wall, the expression of Ephrin ligands in endothelial cells was determined. Again, publicly available data were combined and showed that endothelial cells highly expressed all Ephrin ligands except *EphrinA2*, which is only moderately expressed. Most highly expressed were the ligands *EphrinA1* and *EphrinB2* (Fig. 2A). Since the EPHB2 receptor has the highest binding affinity for the ligands EphrinB1 and EphrinB2^23^ and an increase in endothelial mRNA expression of *EphrinB1* was observed when cells were exposed to pro‐atherosclerotic conditions (Fig. 2B), the atherosclerotic lesions were also stained for EphrinB ligands. We observed EphrinB expression in normal aortic tissue without atherosclerotic plaques (Fig. 2C and D, Stage I). When plaques at different stages were present, the expression of EphrinB remained relatively constant. However, at the stage of a calcified fibrous plaque, the expression of EphrinB increased (1.6‐fold, Fig. 2C and D, Stage IV). As EphrinB expression was not specific for endothelial cells and EphrinB ligands were also expressed in leukocytes and vascular smooth muscle cells (Figs. 1A and 2A), a region‐specific quantification was performed to explore the expression in the different cell types within the plaques. The increase in EphrinB expression was mainly observed in the intima and primarily in the most severe disease state (Fig. 2E). In addition, when looking specifically to the plaque area, an increase in EphrinB expression was observed (Fig. 2F).

### 3.2 EPHB2‐induced reverse signaling has no effect on endothelial barrier function

**FIGURE 3:**
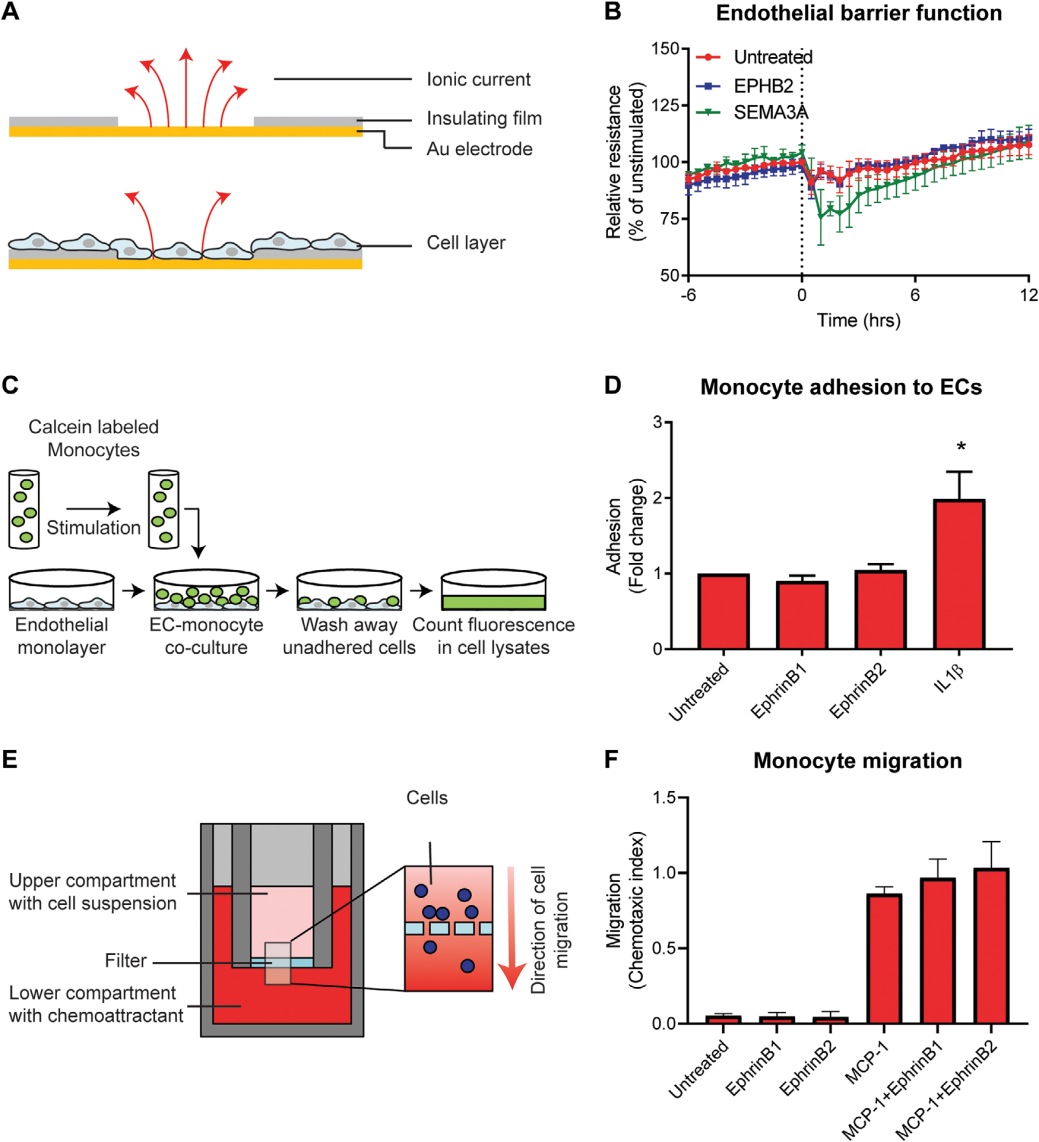
**Induced EPH‐Ephrin signaling has no effect on endothelial barrier function, monocyte migration, and adhesion**. (**A**) Schematic overview of the ECIS system, where changes in resistance by, for example, adherent cells are measured. (**B**) Transendothelial electrical resistance of HUVECs cultured on ECIS electrodes treated with EPHB2, SEMA3A (positive control), or vehicle (untreated) at *t* = 0. Barrier function is represented as percentage resistance of unstimulated HUVECs at time point 0. Mean ± sem of *n* = 3. (**C**) Schematic overview of the adhesion assay. (**D**) Quantification of adhesion of unstimulated THP1 cells, THP1 cells stimulated with recombinant EphrinB1/B2 (500 ng/ml) or the positive control IL‐1β (20 ng/ml). Results are presented relative to unstimulated cells, set as 1. Mean ± sem of *n* = 3. ^*^ *P* < 0.05. (**E**) Schematic overview of the Boyden chamber assay. (**F**) Quantification of migration of THP1 cells toward MCP‐1 (10 ng/ml) alone or combined with EphrinB1/B2. Data are presented as relative to unexposed cells, set as 1. Mean ± sem of *n* = 3

Based on the increased EphrinB expression in atherosclerotic plaques and in endothelial cells upon exposure to pro‐inflammatory cytokines (Fig. 2), we hypothesized that monocyte binding to the endothelium induces EphrinB reverse signaling, resulting in an altered endothelial barrier function. To investigate the role of potential reverse EphrinB signaling in endothelial cells, we added recombinant EPHB2 protein to endothelial cells and assessed the endothelial barrier function by measuring electrical resistance with ECIS (Fig. 3A). No difference in barrier function of the endothelial monolayer was observed when EphrinB reverse signaling was induced by addition of EPHB2 (Fig. 3B). Higher or lower concentrations of EPHB2 also did not alter barrier function (Supplementary Fig. 3A), while induction of Semaphorin3A signaling did result in a decrease in barrier function (Fig. 3B).

### 3.3 EphrinB‐induced forward signaling has no influence on monocyte trafficking

In addition to the role of reverse signaling on endothelial barrier function, we assessed the role of EphrinB1‐ and EphrinB2‐induced forward signaling on monocyte adhesion and migration. Monocytes were stimulated with recombinant EphrinB1 or EphrinB2 for 30 min before adding them to a confluent monolayer of endothelial cells (Fig. 3C). Both EphrinB1 and EphrinB2 stimulation did not change the adhesion ability of the monocytes, while stimulation with IL‐1β did induce monocyte adhesion (Fig. 3D and Supplementary Fig. 3B). Next, using the Boyden chamber assay (Fig. 3E), monocyte migration toward MCP‐1 in the presence or absence of EphrinB1 or EphrinB2 was examined. We observed that both EphrinB1 and EphrinB2 in the absence of MCP‐1 had no chemoattractant effect on the monocytes, nor did EphrinB1 or EphrinB2 in combination with MCP‐1 had an antagonistic effect on monocyte chemotaxis (Fig. 3F).

### 3.4 EPHB2 on monocytes promotes monocyte adhesion and migration

**FIGURE 4:**
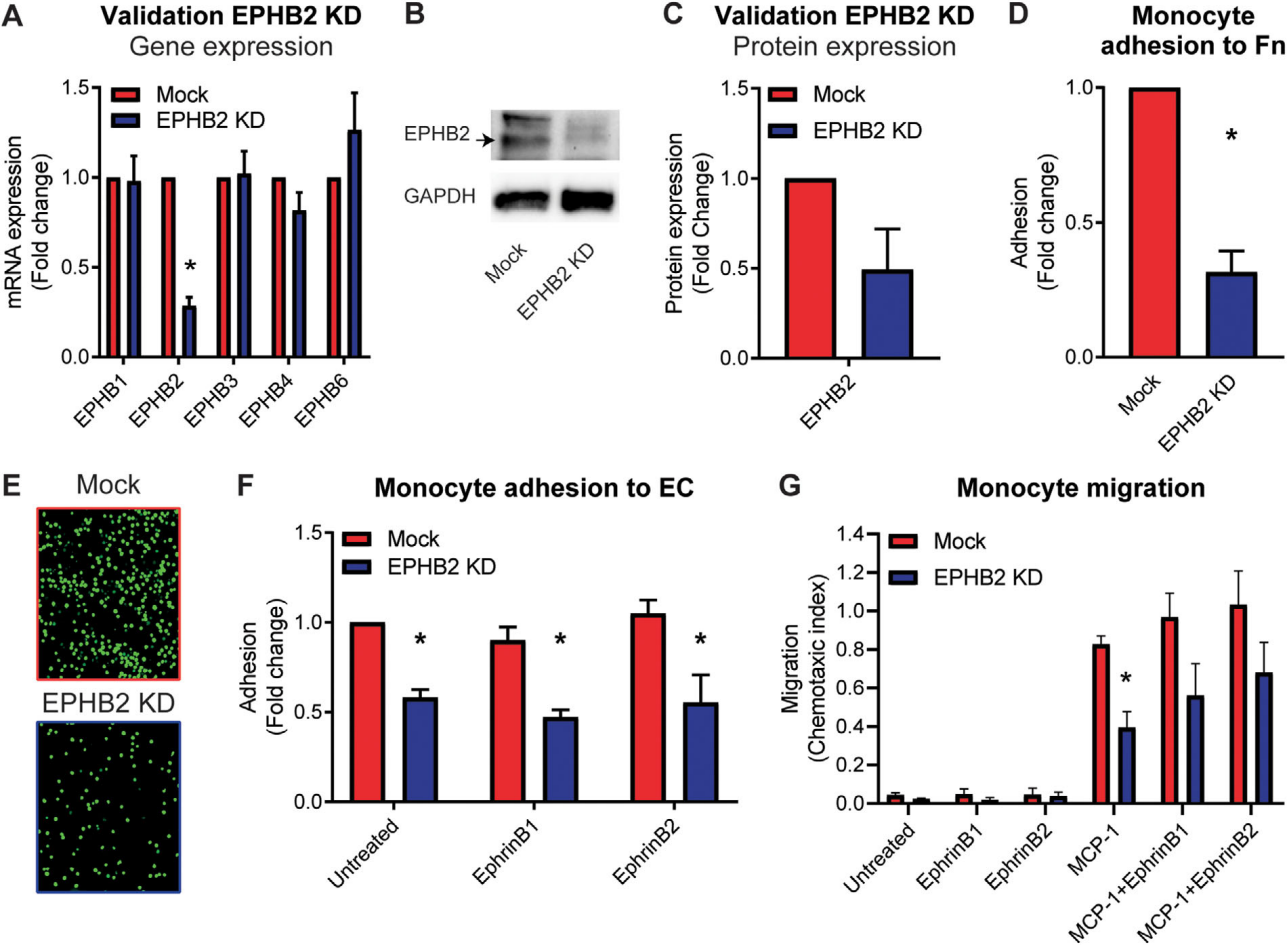
**Loss of EPHB2 on monocytes reduces monocyte adhesion and migration**. (**A**) mRNA expression of *EPHB1/B2/B3/B4/B6* in mock control monocytes or monocytes treated with a shRNA against *EPHB2*. Results are relative to mock control cells, set as 1. Mean ± sem of *n* = 3. ^*^ *P*  <  0.05. (**B** and **C**) Immunoblots (**B**) and quantification (**C**) of EPHB2 expression in mock and EPHB2 knockdown THP1 cells. Expression is corrected for GAPDH and expressed as fold change compared to mock THP1 cells, set as 1. Mean ± sem of *n* = 4. (**D–F**) Quantification of adhesion of mock and EPHB2 knockdown THP1 cells to fibronectin‐coated wells (**D**) or to HUVECs, either unstimulated or stimulated with EphrinB1/B2 (500 ng/ml) (**E** and **F**). Results are relative to untreated control cells, set as 1. Mean ± sem of *n* = 15 or *n* = 3, respectively. ^*^ *P* < 0.05. (**G**) Migration of mock cells and EPHB2 knockdown THP1 cells to MCP‐1 (10 ng/ml) alone or combined with EphrinB1/B2. Mean ± sem of *n* = 3

As a ligand‐dependent effect for EPHB2 could not be confirmed, we tested the ligand‐independent potential of EPHB2 on monocyte adhesion and migration. For this, EPHB2 expression in THP1 cells was silenced using a lentiviral shRNA targeting *EPHB2* mRNA. A non‐*EPHB2* targeting scrambled shRNA was used as a control. Repression of EPHB2 was validated using qPCR and immunoblot, showing a decrease by ≈75% on mRNA level and ≈40% on protein level (Fig. 4A–C). Adhesion of these THP1 cells to either fibronectin or to a confluent monolayer of HUVECs was diminished compared to control THP1 cells (Fig. 4D–F). Stimulation of THP1 cells with EphrinB1 or EphrinB2 before adhesion to HUVECs did not result in differences in adhesion capacity of the EPHB2 knockdown THP1 cells (Fig. 4F and Supplementary Fig. 3B). In line with the reduced adhesion of EPHB2 knockdown THP1 cells, also migration toward MCP‐1 was decreased in the EPHB2 knockdown THP1 cells compared to control cells, which was not influenced by the addition of EphrinB1 or EphrinB2 (Fig. 4G).

### 3.5 EPHB2 affects actin cytoskeleton via phosphorylation of FAK

**FIGURE 5:**
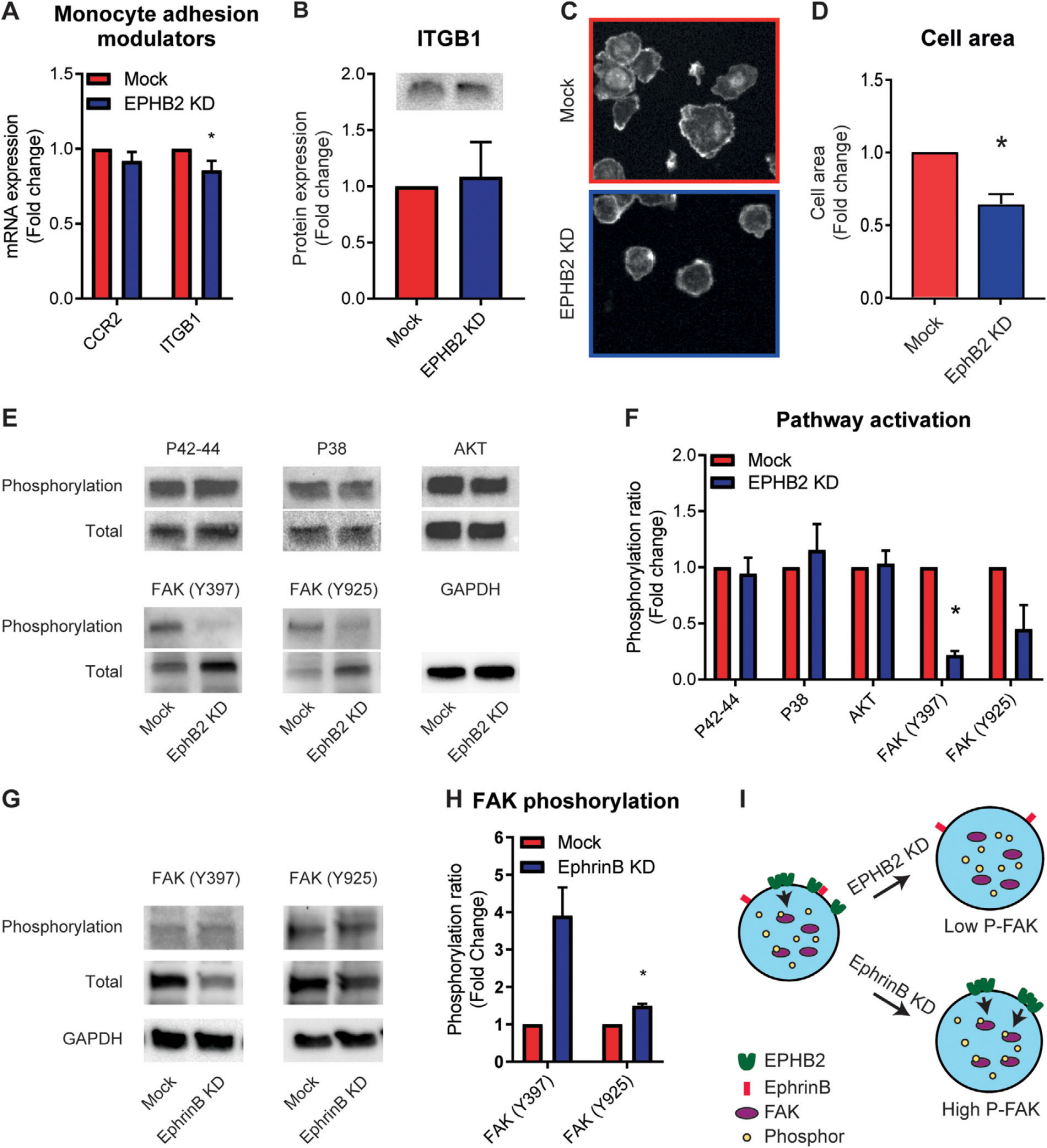
**Reduced cell spreading and decreased phosphorylation of FAK in THP1 cells with decreased expression of EPHB2**. (**A**) *CCR2* and *ITGB1* expression in control and EPHB2 knockdown monocytes. Results are relative to mock control cells, set as 1. Mean ± sem of *n* = 3. ^*^ *P* < 0.05. (**B**) Quantification of ITGB1 immunoblot expressed as fold change compared to control THP1 cells and corrected for GAPDH. (**C** and **D**) Representative pictures (**C**) and quantification (**D**) of the cell surface area of adhered control and EPHB2 knockdown THP1 cells. Results are relative to mock control cells, set as 1. Mean ± sem of *n* = 3. ^*^ *P* < 0.05. (**E** and **F**) Immunoblots (**E**) and quantification (**F**) of phospho‐ and total P38, P42‐44, AKT, FAK (Y397 and Y925), and GAPDH in control cells and EPHB2 knockdown cells. (**G** and **H**) Immunoblots (**G**) and quantification (**H**) of phospho‐ and total FAK (Y397 and Y925) and GAPDH in control cells and EphrinB knockdown cells. (**F** and **H**) Expression is expressed as fold change compared to mock THP1 cells and is corrected for total protein expression. Mean ± sem of *n* = 3. ^*^ *P* < 0.05. (**I**) Schematic diagram of the effect of EPHB2 and EphrinB expression on FAK phosphorylation in monocytes

To explain the reduced adhesion and migration of THP1 cells with reduced levels of EPHB2, we hypothesize that this could be mediated by changes in the expression levels of the MCP‐1 receptor C‐C chemokine receptor 2 (CCR2) or the main binding integrin of monocytes Integrinβ‐1 (ITGB1). mRNA expression of *CCR2* did not differ in EPHB2 knockdown cells compared to mock treated cells while mRNA levels of *ITGB1* were slightly, but significantly decreased (Fig. 5A). Despite the observed moderate decrease in *ITGB1* on mRNA level, no change was observed in protein expression of ITGB1 (Fig. 5B). Visualization of the cells revealed a more rounded morphology and smaller cell area upon adhesion in monocytes with reduced expression of EPHB2 compared to control monocytes (Fig. 5C and D). Based on this observation polarization of the monocytes was evaluated by determining expression levels of the inflammatory Mϕ (M1) markers TNF‐α, IL‐1β, IL‐6, and CD86 and the anti‐inflammatory Mϕ (M2) markers IL‐10 and CD163 in monocytes and monocyte‐derived macrophages with or without a knockdown of EPHB2. While differences in marker expression were observed between monocytes and macrophages, no clear differences in expression between mock control and EPHB2 knockdown cells were observed (Supplementary Fig. 4A and B). Next, cellular pathways involved in regulation of the cytoskeleton, such as phosphorylation of the MAPK pathway (p42‐44 and p38), protein kinase B (AKT) and focal adhesion kinases (FAK) were evaluated. While phosphorylation of p42‐44, p38, and AKT were comparable between EPHB2 knockdown and mock control cells, we observed a significant decrease in phosphorylation of FAK at Y397 in EPHB2 knockdown cells. Phosphorylation of FAK at Y925 was also lower in EPHB2 knockdown cells, but this difference did not reach statistical significance (Fig. 5E and F). We next investigated whether phosphorylation of FAK via EPHB2 was entirely ligand‐independent and not caused by a *cis*‐interaction between the EPHB2 receptor and its EphrinB ligands on the same cell. From the EphrinB ligands, monocytes highly expressed EphrinB1 (Fig. 1A). We therefore transduced THP1 cells with a lentiviral shRNA targeting EphrinB1 mRNA. Gene and protein analysis showed a significant reduction in EphrinB1 expression and a slight reduction of EphrinB2 (Supplementary Fig. 5A–C). In these cells, we observed an increase in phosphorylation of FAK upon knockdown of EphrinB ligands compared to control cells (Fig. 5G and H). Together with the reduced FAK phosphorylation upon EPHB2 knockdown, this suggests that activation of FAK via EPHB2 most likely occurs via receptor dimerization and is independent of its ligand in both *cis‐* and *trans‐*interactions (Fig. 5I).

## 4 DISCUSSION

It is acknowledged that Ephrin family members are involved in atherosclerotic related processes, like among others leukocyte chemotaxis, adhesion, and migration, and regulation of atherosclerotic inflammation.^12^, ^13^, ^14^, ^24^ This is not surprising since EPHA2, EPHA8, and EPHB2, are located within the murine Athsq1 atherosclerosis susceptibility locus,^25^ which is highly homologous to the premature myocardial infarction susceptibility locus in human^18^ that similarly contains EPHA2, EPHA8, and EPHB2. Using multiple functional assays, we now show pro‐atherosclerotic functions of EPHB2, since reduced levels of this receptor resulted in less monocyte adhesion and migration via decreased phosphorylation of FAK, suggesting a role for EPHB2 in monocyte accumulation in atherogenesis.

In the current study, we have shown, for the first time to our knowledge, a plaque burden‐dependent expression of EPHB2 and EphrinB ligands in atherosclerotic plaques. In accordance to a paper of Sakamoto and co‐workers, we show that expression of EPHB2 and EphrinB is increased in advanced atherosclerotic plaques,^14^ but we now add that expression of EPHB2 proportionally increases with plaque burden. Most of the EPHB2 was found in cells of the monocyte/Mϕ lineage, while an increase in EphrinB ligand was mainly observed in the intima of the vessel wall where also the endothelial cells reside. However, this layer is often disrupted and/or damaged during atherosclerotic plaque progression and we also observed an increase of expression within the plaque area itself, indicating that EphrinB expression is not specific to endothelial cells but also present in other cells, for example, macrophages, smooth muscle cells, and T lymphocytes.

Ephrins and their receptors are expressed on both endothelial cells and leukocytes. It is therefore not surprising that they are involved in monocyte‐endothelial interactions. Forward and reverse signaling of the monocytic EPHB receptors and endothelial EphrinB ligands are on one end proposed to stimulate monocyte adhesion and transmigration, while on the other end to reduce the barrier of endothelial monolayers.^26^, ^27^, ^28^ Despite the increase of both EPHB2 and EphrinB ligands in plaque tissue, we were not able to delineate the exact role for this receptor‐ligand combination in the monocyte‐endothelial interactions important for atherogenesis in our in vitro experiments. Not only did activation of endothelial EphrinB ligands, by exposing them to recombinant EPHB2 receptor, have no effect on endothelial barrier function but also activation of EPH receptors on monocytes with recombinant EphrinB ligands had no effect on monocyte adhesion and little effect on migration. While our results seem to be in difference with other studies that have shown that both EphrinB1 and EPHB2 can inhibit the migration of monocytes,^14^ it should be noted that the means of Ephrin ligand presentation is of importance. While a surface coated with Ephrin ligands repels leukocyte migration,^14^, ^29^ soluble EphrinB ligand in a Transwell system promotes migration of primary blood mononuclear cells.^10^, ^30^ Our study adds that EphrinB1/B2 present in the lower compartment of a Transwell system had no chemo‐attractive effect on THP1 monocytes and that stimulation with soluble EphrinB1 or EphrinB2, did not influence the adhesion capacity of monocytes.

However, our in vitro data did show that knockdown of EPHB2 in monocytes resulted in impaired adhesion and migration of these monocytes compared to control monocytes. Again, the presence of either EphrinB1 or EphrinB2 did not change monocyte adhesion and slightly increased monocyte migration but this effect was independent of the presence of the EPHB2 receptor on monocytes. Since EphrinB1 and EphrinB2 do not solely bind to EPHB2,^7^ other receptor‐ligand interactions may have resulted in this effect. Taken together, these results suggest a ligand‐independent function of EPHB2 in monocyte adhesion and migration.

The actin cytoskeleton is known to be important for monocyte cellular shape and can thereby influence monocyte adhesion and migration. We indeed observed that EPHB2 knockdown monocytes adopt a specific and rounded shape preventing cellular adhesion and cell spreading. It is known that FAKs are important regulators in cell remodeling and cell migration. Phosphorylation of FAK at Tyr397 (Y397) results in binding and activation of Src protein tyrosine kinases, which then can activate for example small GTPases, and thereby regulate the cells actin cytoskeleton and cellular migration. Earlier studies have already implicated a role for EPH‐Ephrin signaling, mainly EPHA2, and focal adhesion kinases in regulation of the actin cytoskeleton.^31^, ^32^ Moreover, Batlle et al. showed in colon epithelial tumor cells that stimulation with EphrinB1 results in reduced FAK activation.^33^ In our study, we have shown that monocytes with diminished EPHB2 expression have less phosphorylation of FAK Tyr397 explaining the reduced cell spreading, adhesion, and migration capacity of these cells. No significant changes were observed in the FAK phosphorylation at Tyr925 (Y925), which is in line with unchanged MAPK phosphorylation as FAK phosphorylation at Tyr925 is linked to the activation of the extracellular signal regulated MAPK pathway.^34^ Due to the complex nature of EPH receptor interactions, it is not surprising that EPH receptors can function independent of its ligand. Earlier studies have for example shown that unstimulated EPHA2 receptors are constitutively associated with FAK.^35^ This was followed by studies of Barquilla et al. and Miao et al. indicating ligand‐independent regulation of EPHA2 signaling.^36^, ^37^ To complicate it even further, EPH receptors can also signal in a lateral *cis* interaction between EPH receptors and Ephrins on the same cell,^38^, ^39^ inducing forward signaling within the same cell. For EPHB2, a ligand‐independent function has not been shown before. As monocytes highly express EphrinB1 and EphrinB1 has a high affinity for the EPHB2 receptor^7^ a *cis* interaction in monocytes could occur. However, our result that monocytes with a knockdown in EphrinB ligand do not have a decreased, as would be expected in loss of *cis* interaction‐induced signaling, but an increased activity of FAK renders it highly unlikely for an EPHB2‐EphrinB *cis* interaction to regulate FAK activity in monocytes. The observed increase in FAK activity upon EphrinB knockdown might be explained by more unbound EPHB2 receptor present on the cell membrane allowing for EPHB2 dimerization and phosphorylation of FAK. Taken together, we propose a mechanism by which EPHB2 on monocytes without ligand, neither in a *trans‐* nor a *cis‐*interaction, associates with FAK and thereby promotes Y397 phosphorylation, cellular remodeling, and migration. With the knockdown of EPHB2 the constitutive phosphorylation of FAK is no longer present and the monocytes’ actin cytoskeleton is deregulated inhibiting its migration.

As mentioned before, limited in vivo data on the role of EPHB2 in atherosclerosis is available. EPHB2 expression in vivo is not limited to monocytes and is expressed in a broad range of other cell types including, for example, neurons, T cells, and intestinal (progenitor) cells.^16^ In line with the variety of cells that express EPHB2, there is also a wide range of functions known for EPHB2 ranging from axonal and vascular patterning during development^40^ to, for example, regulating cellular invasiveness of cancer cells.^41^ So even though general EPHB2 knockout mice are viable and available, research seems to be holding back because of the broad range of potent and essential biological functions of Ephrins during development and in (pathological) physiology. However, specific knockout of EPHB2 in the monocytic cell lineage would be an interesting way to further study the role of monocytic EPHB2 in atherosclerosis in vivo.

In line with the limited amount of in vivo studies, to date also no clinical studies have been conducted on the potential therapeutic options of EPHB2 and its EphrinB ligands in inflammation, immunity, or atherosclerosis. In cancer research several clinical trials are conducted with different EPH receptor targeting agents,^42^ but for EPHB2, clinical trials are still awaiting. Despite some promising results with a drug‐conjugated Ab raised against EPHB2, which is expressed in melanomas, neuroblastomas, gastric, lung, and colon cancers,^43^, ^44^, ^45^, ^46^ which could induce cell death in EPHB2 expressing cells both in vitro and in vivo,^47^ no clinical data has been reported yet. Whether this Ab will be a useful treatment option needs to be further investigated and discovering its therapeutic potential might even guide the way for its implication in other diseases like, for example, atherosclerosis.

Since we have shown in this study that lowering the expression of EPHB2 on monocytes inhibits monocyte adhesion and migration, cell specific targeting of EPHB2 remains a promising potential therapeutic target for atherosclerotic disease. The upcoming field of antisense oligonucleotides (ASOs)^48^ might in time provide opportunities to specifically deliver ASOs raised against EPHB2 to inflamed regions and thereby reduce subendothelial monocyte accumulation. However, these options are still far from clinically relevant and further exploration of not only the ASOs but also EphrinB and EPHB2 is essential for discovering new therapeutic options.

In summary, the present study demonstrates an increased expression of EphrinB and EPHB2 in progressive human atherosclerotic tissue. Although the exact means by which Ephrins affect atherosclerosis development remains to be elucidated, we have shown that EPHB2 plays a role in atherosclerosis by mechanisms that are not related to the activation by *trans* nor *cis* interaction of the currently known EphrinB ligands. We show that the effect of EPHB2 is partially explained by its effect on FAK phosphorylation. The EPHB2 receptor‐induced increase in FAK phosphorylation results in a cytoskeletal rearrangement, rendering the monocytes more prone to adhere, spread, and migrate through the endothelial cell layer, which could contribute to monocyte/Mϕ accumulation and progression of atherosclerosis.

## AUTHORSHIP

D.V. and C.B. contributed equally to this paper. D.V., C.B., H.Z., G.K.H., A.v.Z., and J.v.G. conceptualized and designed the study and contributed to analysis and interpretation. D.V., C.B., S.C., R.L., A.K., and J.v.G. performed the data collection. D.V. and C.B. drafted the paper. G.K.H. and J.v.G. revised the work and approved the final version of the manuscript. D.V. and C.S.B. contributed equally to this work.

## DISCLOSURE

G.K.H. has served as consultant and speaker for biotech and pharmaceutical companies that develop molecules that influence lipoprotein metabolism, including Regeneron, Pfizer, MSD, Sanofi, and Amgen. Until April 2019, G.K.H. has served as PI for clinical trials conducted with A.O. Amgen, Sanofi, Eli Lilly, Novartis, Kowa, Genzyme, Cerenis, Pfizer, Dezima, Astra Zeneca. The Department of Vascular Medicine receives the honoraria and investigator fees for sponsor studies/lectures for companies with approved lipid lowering therapy in the Netherlands. Since April 2019, G.K.H. is partly employed by Novo Nordisk (0.7FTE) and the AMC (0.3FTE). G.K.H. has no active patents nor share or ownership of listed companies. The other authors declare that the research was conducted in the absence of any commercial or financial relationships that could be construed as a potential conflict of interest.

## Supporting information

Supporting Information
