# Supplementary material for: EPH receptor B2 stimulates human monocyte adhesion and migration independently of its EphrinB ligands
Source: J Leukoc Biol. 2020 Apr 26;108(3):999–1011. doi: 10.1002/JLB.2A0320-283RR (PMC7496365; doi:10.1002/JLB.2A0320-283RR)
Supplement: Supplementary file 1 — Supporting Information [file JLB-108-999-s001.pdf]

Supplemental table and figures

EPH Receptor B2 stimulates human monocyte adhesion and migration independently of its EphrinB ligands

Dianne Vreeken [1]*, Caroline Suzanne Bruikman [1]†, Stefan Martinus Leonardus Cox*, Huayu Zhang*, Reshma Lalai*, Angela Koudijs*, Anton Jan van Zonneveld*, Gerard Kornelis Hovingh†, Janine Maria van Gils*

[1] Authors contributed equally to this paper

* Leiden University Medical Centre, Department of Internal Medicine, Einthoven Laboratory for Vascular and Regenerative Medicine, Leiden, The Netherlands

† Amsterdam UMC, University of Amsterdam, Department of Vascular Medicine, Amsterdam Cardiovascular Sciences, Meibergdreef 9, Amsterdam, The Netherlands

Supplemental TABLE 1 Primer sequences.

**Gene**	**Forward sequence**	**Reverse sequence**
GAPDH	CCTGCACCACCAACTGCTTA	GGCCATCCACAGTCTTCTGAG
EphrinB1	GAGGCAGACAACACTGTCAAG	AGCTTCAGTAGTAGGACCGTC
EphrinB2	TGTGGGTATAGTACCAGTCTTG	ACTGCTGGGGTGTTTTGATGG
EphrinB3	TCGGCGAATAAGAGGTTCCA	GTCCCCGATCTGAGGGTACA
EPHB1	TACGGCAAGTTCAGTGGCAA	AGGACACAACGAACACGACC
EPHB2	GCTTCGAGGCCGTTGAGAAT	GAAGTGGTCCGGCTGTTGAT
EPHB3	GTCATCGCTATCGTCTGCCT	AAACTCCCGAACAGCCTCATT
EPHB4	CGCACCTACGAAGTGTGTGA	GTCCGCATCGCTCTCATAGTA
EPHB6	CGACCAGACCAATGGGAACA	GGGTGAAGGAGTGGGATTCG
CCR2	CCACATCTCGTTCTCGGTTTATC	CAGGGAGCACCGTAATCATAATC
ICAM-1	GGCCGGCCAGCTTATACAC	TAGACACTTGAGCTCGGGCA
TNFα	CCTCTCTCTAATCAGCCCTCTG	GAGGACCTGGGAGTAGATGAG
IL1β	ATGATGGCTTATTACAGTGGCAA	GTCGGAGATTCGTAGCTGGA
IL6	AAGCCAGAGCTGTGCAGATGAGTA	AACAACAATCTGAGGTGCCCATGC
IL10	GCGCTGTCATCGATTTCTTCC	GTAGATGCCTTTCTCTTGGAGCTTA
CD86	CTGCTCATCTATACACGGTTACC	GGAAACGTCGTACAGTTCTGTG
CD163	TTTGTCAACTTGAGTCCCTTCAC	TCCCGCTACACTTGTTTTCAC

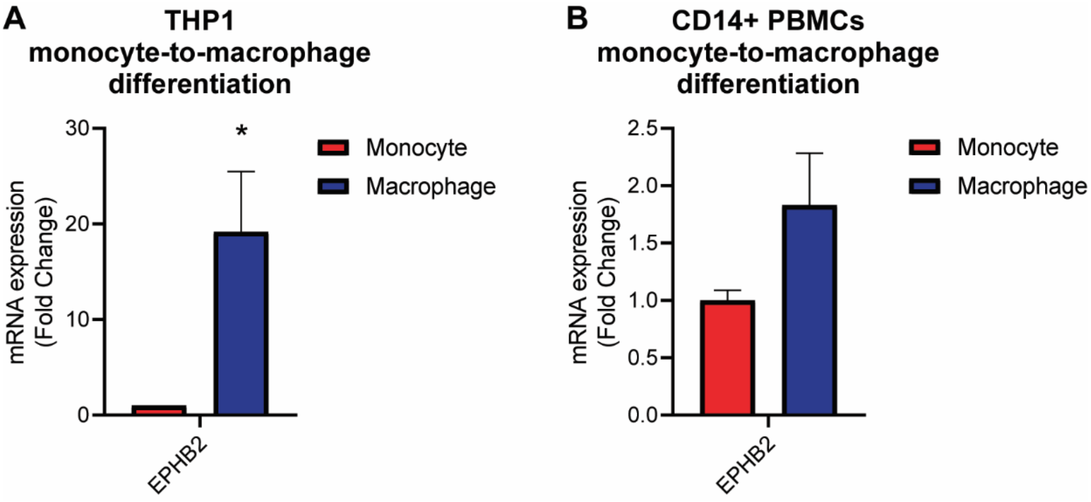

Supplemental figure 1 Increased EPHB2 expression upon monocyte-to-macrophage differentiation.

mRNA expression of EPHB2 in monocytes or macrophages derived from (**A**) THP1 cells or (**B**) CD14+ derived peripheral blood mononuclear cells. Results are relative to monocytes, set as 1. Mean ± s.e.m. of n=3. *P < 0.05.

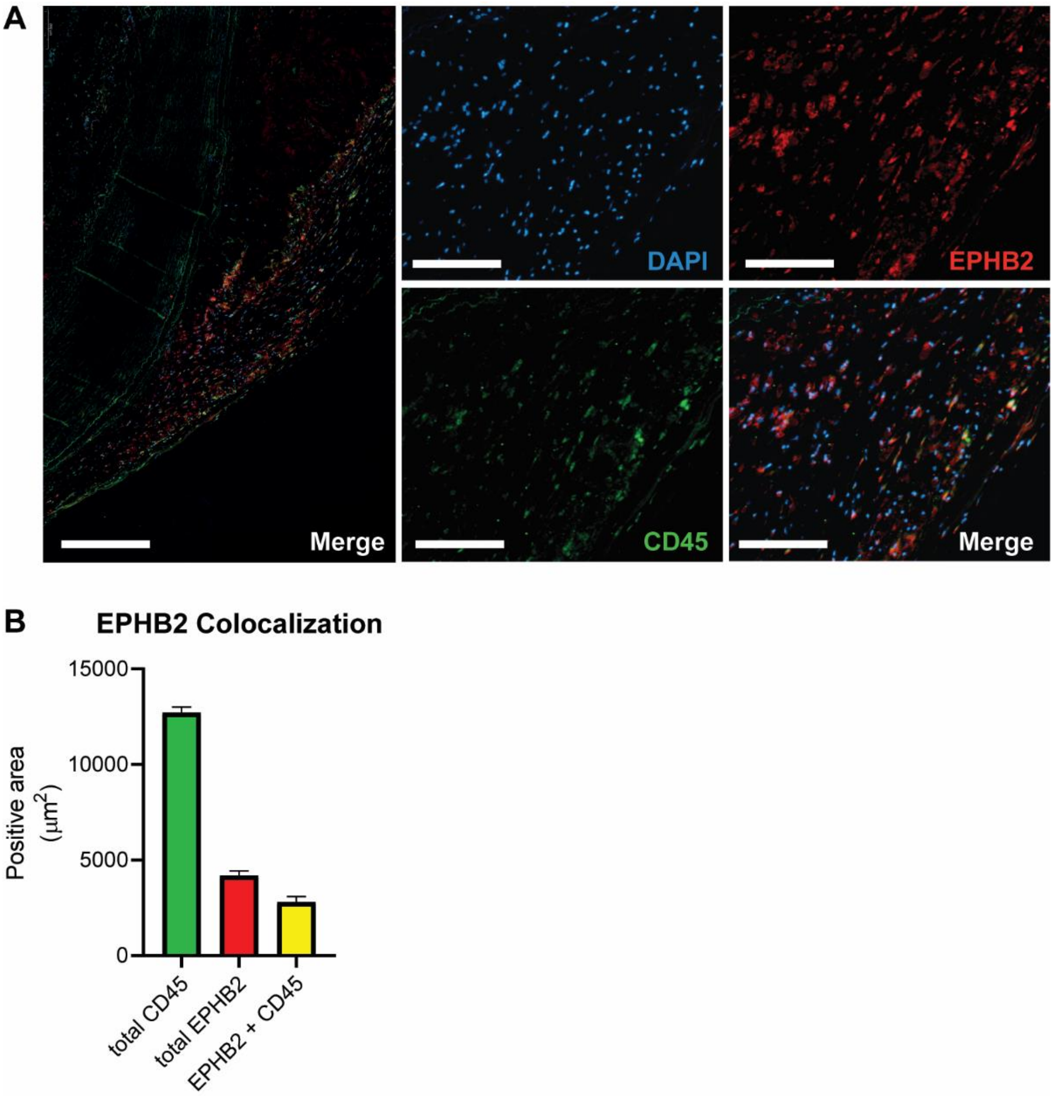

Supplemental figure 2 EPHB2 colocalization with CD45.

(**A**) Overview and zoom-in pictures of immunofluorescent staining for EPHB2 (red), CD45 (green) and nuclei (blue) in stage IV human aortic sections. Scale bars represent 300 and 25 µm respectively. (**B**) Quantification of fluorescent signal in plaque shoulder region. Results are quantified as positive area in μm^2. Mean ± s.e.m of n=6.

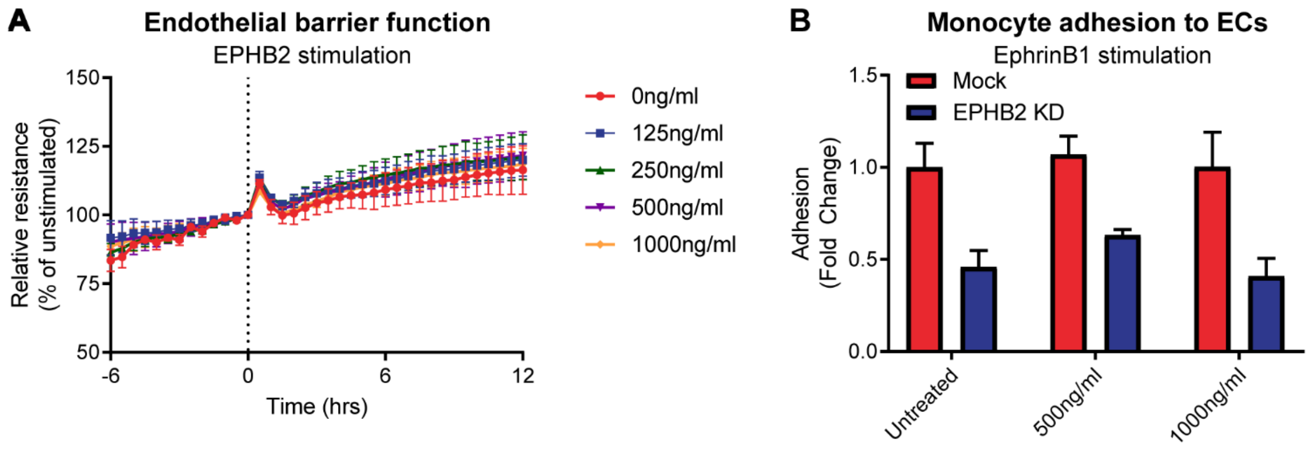

Supplemental figure 3 Different concentrations of recombinant protein did not alter cellular response.

(**A**) Transendothelial electrical resistance of EPHB2 stimulated HUVECs cultured on ECIS electrodes. Concentrations of EPHB2 ranged from 0 to 1000ng/ml. Barrier function is represented as percentage resistance of unstimulated HUVECs at time point 0. Mean ± s.e.m. of n=3. (**B**) Adhesion of unstimulated THP1 cells or THP1 cells stimulated with different concentrations of recombinant EphrinB1 (500 or 1000 ng/ml). Results are presented relative to unstimulated cells, set as 1. Mean ± s.e.m. of triplicate samples.

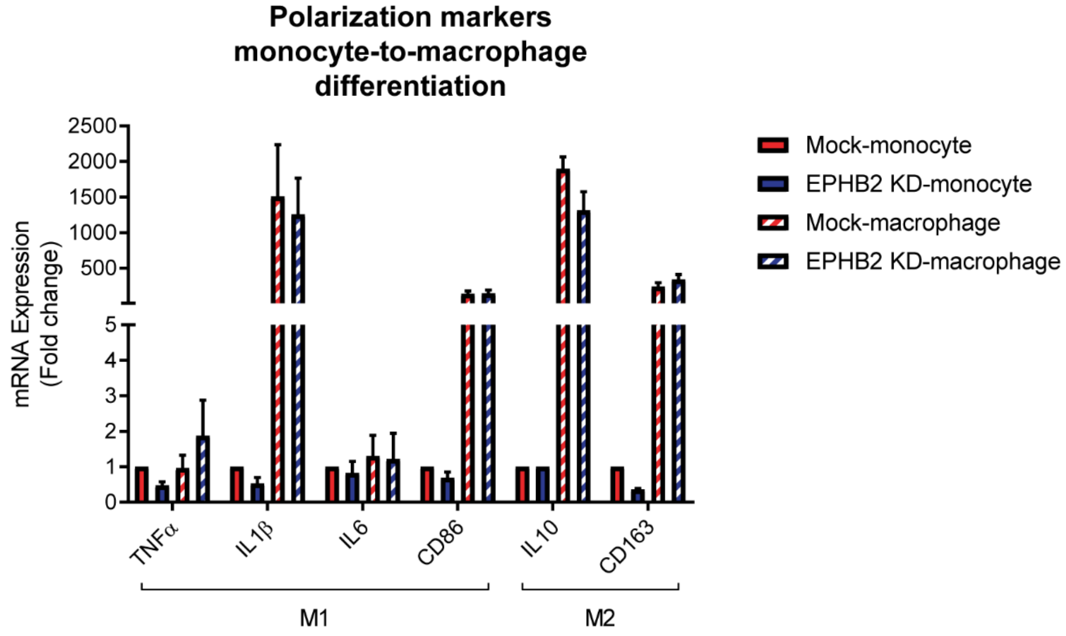

Supplemental figure 4 No altered monocyte/macrophage polarization upon EPHB2 knockdown. mRNA expression of M1 markers TNFα, IL1β, IL6 and CD86 and M2 markers IL10 and CD163 in monocytes (solid filled bars) or macrophages (striped bars) derived from mock control THP1 cells or THP1 cells with a knockdown in EPHB2. Results are relative to mock control monocytes, set as 1. Mean ± s.e.m. of n = 3.

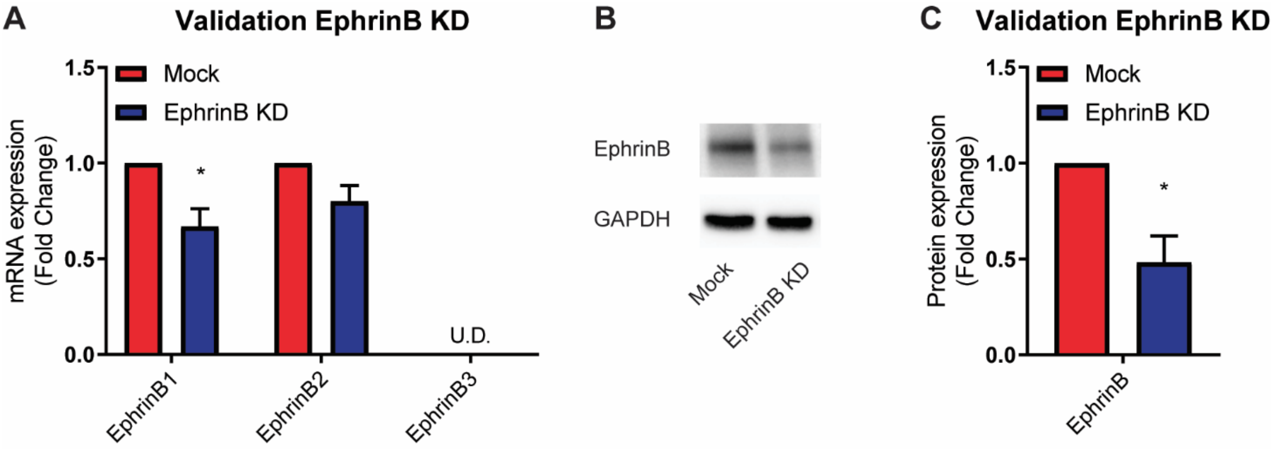

Supplemental figure 5 Decreased EphrinB expression in shRNA transduced THP1 cells. (**A**) mRNA expression of EphrinB ligands in mock control monocytes or EphrinB knockdown monocytes. Results are relative to mock control cells, set as 1. Mean ± s.e.m. of n=3. *P < 0.05 (**B**) Immunoblots and (**C**) quantification of EphrinB expression in mock and EphrinB knockdown THP1 cells. Expression is corrected for GAPDH protein expression and expressed as fold change compared to mock THP1 cells, set as 1. Mean ± s.e.m. of n=3. *P < 0.05.
